# Strategies for indica rice adapted to high-temperature stress in the middle and lower reaches of the Yangtze River

**DOI:** 10.3389/fpls.2022.1081807

**Published:** 2023-01-06

**Authors:** Man Zhang, Zhong Li, Kaixuan Feng, Yalan Ji, Youzun Xu, Debao Tu, Bin Teng, Qiumeng Liu, Jingwen Liu, Yongjin Zhou, Wenge Wu

**Affiliations:** ^1^ Rice Research Institute, Anhui Academy of Agricultural Sciences, Hefei, Anhui, China; ^2^ School of Resources and Environment, Anhui Agricultural University, Hefei, Anhui, China

**Keywords:** rice, high temperature, carpet seedling mechanical transplantation, sowing date, growth regulator

## Abstract

High temperatures caused by climate warming severely affect the grain yield and quality of rice. In this study, the rice cultivars Longliangyou Huazhan (LLYHZ) and Quanliangyou 2118 (QLY2118) were selected as the experimental materials for investigation of an optimal cultivation system under high-temperature treatment. In addition, the heat-resistant cultivar Huanghuazhan (HHZ) and heat-sensitive cultivar Huiliangyou 858 (HLY858) were chosen as the experimental materials to study the effects of exogenous plant growth regulators on heat stress responses under high-temperature treatment. The results showed that mechanical transplanting of carpet seedlings and delayed sowing effectively increased the leaf area index and reduced the canopy temperature of LLYHZ and QLY2118. Furthermore, carpet seedling mechanical transplantation and delayed sowing improved grain yield and quality. Spray application of five plant growth regulators revealed that brassinolide and salicylic acid had the strongest effects on significantly improving antioxidant enzyme activities in the panicle, which would reduce the damage caused by the accumulation of reactive oxygen species and enhance plant tolerance of high-temperature stress. In addition, brassinolide and salicylic acid enhanced the percentage of anther dehiscence and percentage seed set. In this study, a set of simplified eco-friendly cultivation techniques for single-season indica rice adaptation to high-temperature stress was established. These results will be of great importance in alleviating the effects of high-temperature stress on rice production.

## 1 Introduction

Global warming is primarily manifested as an increase in average temperature and the frequent incidence of extreme heat (Lobell and Gourdji, 2012). General circulation models estimate an average increase in global surface temperature of approximately 4°C (2.9°C to 5.5°C) (UI Hassan et al., 2021). The trend for climate change in China is essentially consistent with the general trend for global change, but distinct geographical characteristics are evident. The rate of warming in the northern region is significantly greater than that in the southern region, and that in the western region is greater than in the eastern region. The middle and lower reaches of the Yangtze River have frequently experienced extremely high temperatures over a prolonged period. High temperature and the impacts of extreme heat in this area occur mainly from mid-July to early August, with the highest frequency in late July. This coincides with the crucial period for the heading and flowering of mid-season rice in the middle and lower reaches of the Yangtze River. The increase in frequency and intensity of extremely high temperatures will greatly impact rice production (Stephen et al., 2022). Therefore, overcoming the effect of high temperatures on rice during the heading stage is an important focus of agricultural research (Bamagoos et al., 2021).

Heat injury caused by climate warming is a major contributor to the decreases in rice yield and quality (Liu et al., 2021). If the temperature during the vegetative growth period of rice exceeds 35°C, tiller production decreases, the rate of growth in plant height declines, the growth of aboveground and underground plant parts is reduced, and the overall rice development period is shortened. These changes affect the reproductive development of rice, reduce the rate of dry matter accumulation, affect the distribution of dry matter in various organs, and thus severely impact productivity and grain quality (Wassmann et al., 2009). High temperatures in the daytime or the nighttime can affect grain quality. Krishnan et al. (Krishnan et al., 2011) reported that short-term high temperature after flowering adversely affects rice quality and starch granules. In prolonged periods of high temperatures, the degree and frequency of chalkiness are increased, and short-term high-temperature stress reduces amylose accumulation in the grain.

Previous studies have confirmed that high temperature affects the growth of rice during various developmental periods (Yu et al., 2022), especially during the heading stage (Zhang et al., 2021). In response, various cultivation strategies have been adopted in rice production, including changing the planting method (Xing et al., 2017), adjusting the sowing date (Sadras et al., 2015), and spraying plants with chemical regulators (Cao and Hua, 2008). Appropriate rice-planting methods not only improve the soil structure and fertility but also promote grain production (Zhou et al., 2022). Altering the sowing date to adjust the growth period of rice can ensure that the crucial developmental stages coincide with an appropriate temperature and light environment, which is conducive to growth and yield formation (Patel et al., 2019). Spray application of plant growth regulators can increase photosynthetic capacity and promote the synthesis of endogenous hormones to reduce damage from high-temperature stress (Fahad et al., 2016a). With global warming, the frequency and extent of extreme weather are increasing, which has a significant impact on rice production. Farmers frequently choose rice cultivars with yield as their primary consideration and ignore the high-temperature resistance of cultivars; if the sowing date is inappropriate, heading and flowering may coincide with high temperatures. In the face of extremely high temperatures, there is currently a lack of preventative and control measures that can be implemented in rice production. Therefore, it is of considerable importance to establish environmentally friendly, simplified cultivation techniques for single-season indica rice to reduce the impact of high-temperature stress on rice production.

## 2 Materials and methods

### 2.1 Field test

#### 2.1.1 Experimental design

The field experiment was conducted in 2019 at the Guohe Base Test Site in Lujiang County, Anhui Province, China. Temperature differences were achieved by employing two sowing dates to simulate high- and normal-temperature environments. The rice cultivars Longliangyou Huazhan (LLYHZ) and Quanliangyou 2118 (QLY2118), which are suitable for cultivation in the middle and lower reaches of the Yangtze River, were selected as the experimental materials. Two sowing dates (16 and 22 May) and two transplanting methods (manual transplanting and blanket seedling automated transplanting) were applied for a total of eight treatments. Transplanting was performed on 15 June. Three replicate plots, each 200 m^2^ in area, were established for each treatment, comprising a total of 24 plots. To prevent the stacking of fertilizer between different plots, all ridges were covered with black plastic film, and the film was inserted on either side of the ridges to a depth of 20 cm. Nitrogen fertilizer (in the form of urea) was applied to each treatment at a rate of 225 kg N ha^−1^ and the ratio of base fertilizer to tiller fertilizer to panicle fertilizer was 5 : 2 : 3. Phosphate fertilizer (in the form of superphosphate) was applied at a rate of 75 kg P ha^−1^ as a base fertilizer. Potassium fertilizer (in the form of potassium chloride) was applied at a rate of 120 kg K ha^−1^ as a base fertilizer and ear fertilizer in two equal amounts. Base fertilizer, tillering fertilizer, and panicle fertilizer were applied 1 d before transplantation, at early tillering (7 d after transplantation), and at the immature panicle differentiation stage, respectively. Water management during the experiment was conducted as follows: immediately after transplanting, irrigation to a depth of 3−5 cm was carried out, 80% sufficient to drain the field at the seedling stage to reduce ineffective tillering; and 7 d before maturity, the field was drained and sun-dried to facilitate harvesting. Strict control of diseases, pests, and weeds was applied throughout the growth period.

#### 2.1.2 Determination of the leaf area index, meteorological data, and yield

At the heading stage, four holes were sampled in each plot based on the average number of tillers. Plants were divided into stems, leaves, and ears. The leaf area index was determined with an LI-3000 (LI-COR; America) leaf area meter.

The canopy temperature and relative humidity of each treatment was measured with a HOBO MX2301A (HOBO MX2301A ONSET HOBO; America) data logger. The air temperature and relative humidity at the heading stage were measured with a micro weather station.

At the maturity stage, an area of 5.0 m^2^ with uniform growth was selected at the center of each plot to measure grain yield. The grain weight was determined after hygrometric adjustment; the grain water content was measured with a grain moisture meter (PM-8188-A kett; Japan), and then the yield with a water content of 13.5% was calculated. Four adjacent sides of the yield area were cut, with three points on each side. A total of 12 representative plants were placed in a net bag for seed testing. After drying in the shade, the total number of grains per sampling point was counted manually. The mean number of grains per panicle at the sampling point was calculated. All rice plants from one hole were placed in a grain separator to remove the husks and empty grains. Based on the number of filled grains obtained after separation, the percentage seed set of the total number of grains was calculated. Two sets of 1,000 grains were sampled from among the filled grains, and the 1,000-grain weight was measured.

### 2.2 Pot experiment

#### 2.2.1 Experimental design

The pot experiment was reliant on the field experiment. At 15 d before heading in the field experiment, plants with comparable growth tillers were selected, and the plants and rhizosphere soil were dug up from the paddy field and transplanted into pots (height 30 cm and diameter 30 cm). At the heading stage, the plant was labeled and transferred to an artificial climate incubator for treatment for 7 d with a high temperature (37°C) or a normal temperature (32°C; the control).

#### 2.2.2 Determination of grain quality and percentage seed set

The pot-grown plants from the same sampling points were cut and placed in a net bag, dried in a cool place, and then threshed manually. All plants from one sampling point were placed in a grain separator to remove the husks and empty grains. The number of filled grains was determined and the percentage seed set relative to the total number of filled grains was calculated.

The filled grains were dried after harvest and stored indoors for 3 months until the physical and chemical properties were stable. The gel consistency, relative crystallinity, and amylose content of the grains were determined using a PERTON IM9500 near-infrared grain analyzer.

### 2.3 Plant growth regulator control test

#### 2.3.1 Experimental design

A screening test for plant growth regulators was conducted at the Hefei Branch of the National Rice Improvement Center, Anhui Academy of Agricultural Sciences, China, in 2020. The heat-resistant rice cultivar Huanghuazhan (HHZ) and the heat-sensitive cultivar Huiliangyou 858 (HLY858) were used in the pot experiment. The seeds were sown on 14 May and the seedlings were cultivated in the field. On 11 June, seedlings of uniform growth were selected and transferred to plastic buckets (height 30 cm and diameter 30 cm). At 1 d before transplantation, 1.0 g of pure nitrogen, 1.5 g of pure phosphorus, and 0.7 g of pure potassium were applied to each bucket and mixed well as a base fertilizer application. At 7 d after transplantation, 0.4 g of pure nitrogen was applied to each bucket as tillering fertilizer. At the immature panicle differentiation stage, 0.6 g of pure nitrogen and 0.7 g of pure potassium were applied to each bucket as panicle fertilizer. Other management measures were conducted in accordance with the requirements for high-yield rice cultivation. When the panicle head emerged from the flag leaf, tillers or main stems of consistent growth were selected for labeling, and the plants were placed in a greenhouse for treatment with high temperature (37°C) or a normal temperature (32°C; the control). Plant growth regulators [brassinolide (BR), 0.15 mg l^−1^ (Chen et al., 2019); salicylic acid (SA), 500 μmol l^−1^ (Yang et al., 2019); abscisic acid (ABA), 100 µmol l^–1^ (Rezaul et al., 2019); 6-benzylaminopurine (6-BA), 60 mg l^–1^ (Wu et al., 2016); and potassium dihydrogen phosphate (MP), 22.05 mmol l^–1^ (Yang et al., 2019)] were applied as a foliar spray on the first day before high-temperature treatment and the third day after treatment; water was applied as the control. After 5 d of high-temperature treatment, the plants were transferred to a greenhouse at ambient temperature for growth. Once there was no risk of high temperatures outdoors, the plants were moved outside to grow to maturity.

#### 2.3.2 Determination of the percentage anther dehiscence and antioxidant enzyme activity

At noon on the third day of high-temperature treatment, anthers were observed under a stereomicroscope and the percentage dehiscence was calculated as follows: anther dehiscence (%) = (number of fully dehiscent anthers + number of partially dehiscent anthers)/total number of anthers examined × 100.

Fresh spikelets treated at high temperature for 3 d were ground in liquid nitrogen and suspended in 5 ml of precooled phosphate-buffered saline (PBS; 50 mM, pH 7.0). The homogenates were centrifuged at 22,000 × g for 10 min at 4°C, and the supernatant was analyzed for antioxidant enzymes superoxide dismutase (SOD), peroxidase (POD), and catalase (CAT). The activities of SOD, POD, and CAT were determined in accordance with the methods of Nahakpam (Nahakpam and Shah, 2011).

### 2.4 Statistical analysis

Three biological replicates were included for all measurements. The significance of differences among the treatments was statistically analyzed using one-way analysis of variance (ANOVA) followed by the least significant difference (LSD) multiple-range test (*p* < 0.05). The statistical packages OriginPro 8.0 (OriginLab, Northampton, MA, USA) and the Data Processing System (DPS) version 7.05 (Zhejiang University, Hangzhou, China) were used for the statistical analyses.

## 3 Results

### 3.1 Appropriate sowing date to alleviate high-temperature *stress*

**Figure 1:**
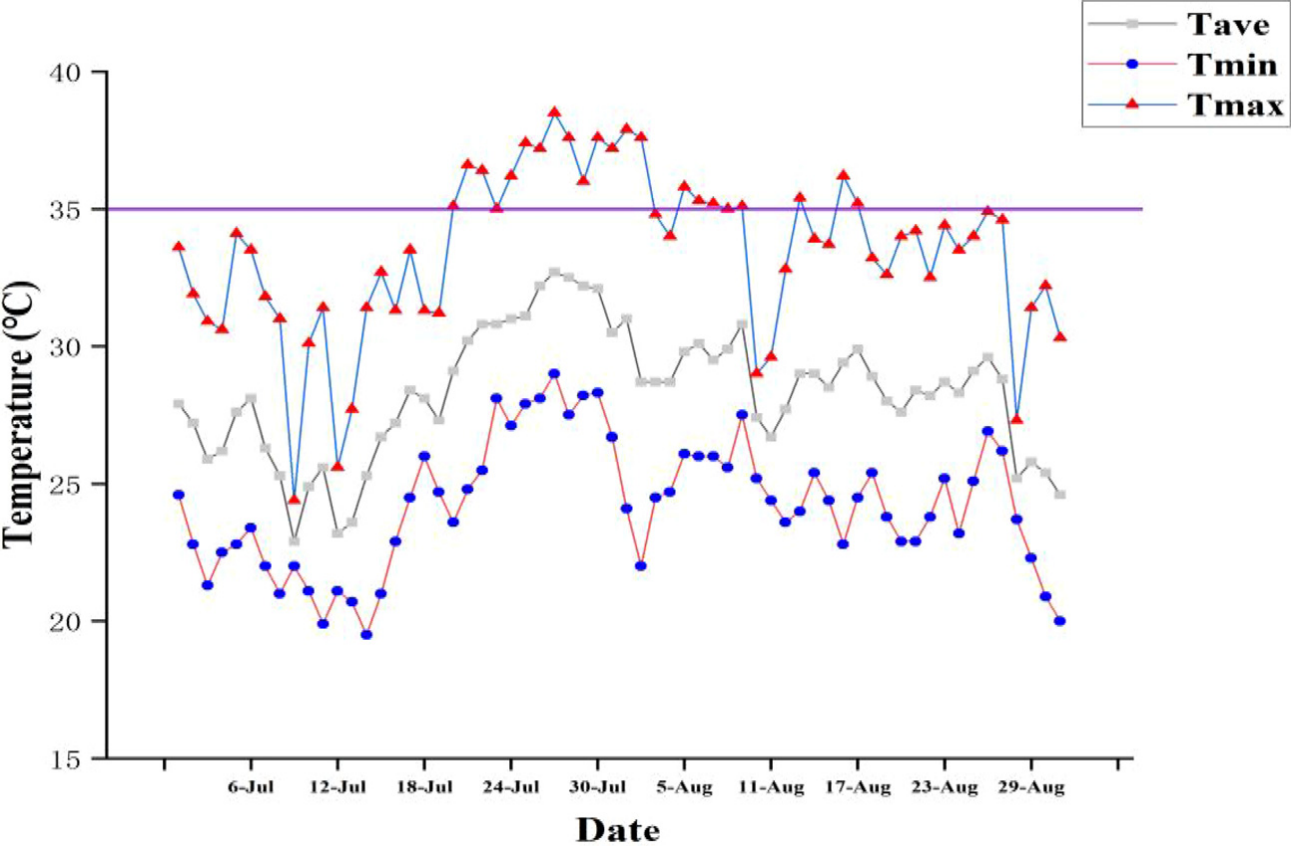
Daily maximum temperature, daily minimum temperature, and daily average temperature in Lujiang district from July to August in 2019. T_ave_, daily average temperature; T_min_, daily minimum temperature; T_max_, daily maximum temperature. The horizontal line is the threshold (35°C) for high temperature.

High temperatures in the Jianghuai area of Anhui Province occur mainly from mid-July to mid-August, with the highest temperatures recorded from late July to early August. In 2019, high temperatures were recorded in late July and early August (in total 13 d) (**Figure 1**). In the present experiment, under the normal sowing date (NSD) (16 May), the heading and flowering period was prone to high-temperature stress. The heading and flowering period of LLYHZ and QLY2118 in the manual transplanting treatment experienced 3 d and 8 d of high temperatures, respectively, whereas in the carpet seedling mechanical transplanting treatment the two cultivars experienced only 1 d and 2 d of high temperatures, respectively. Under delayed sowing (22 May), the number of days of high-temperature stress in the heading and flowering period in the manual transplanting treatment (2 d and 3 d) was lower than that under the normal sowing date, but the plants were still exposed to high-temperature stress, whereas plants in the carpet seedling mechanical transplanting treatment under delayed sowing avoided exposure to high-temperature stress (**Supplementary Table S1**).

### 3.2 Leaf area index and canopy temperature of rice plants at the heading stage

**Figure 2:**
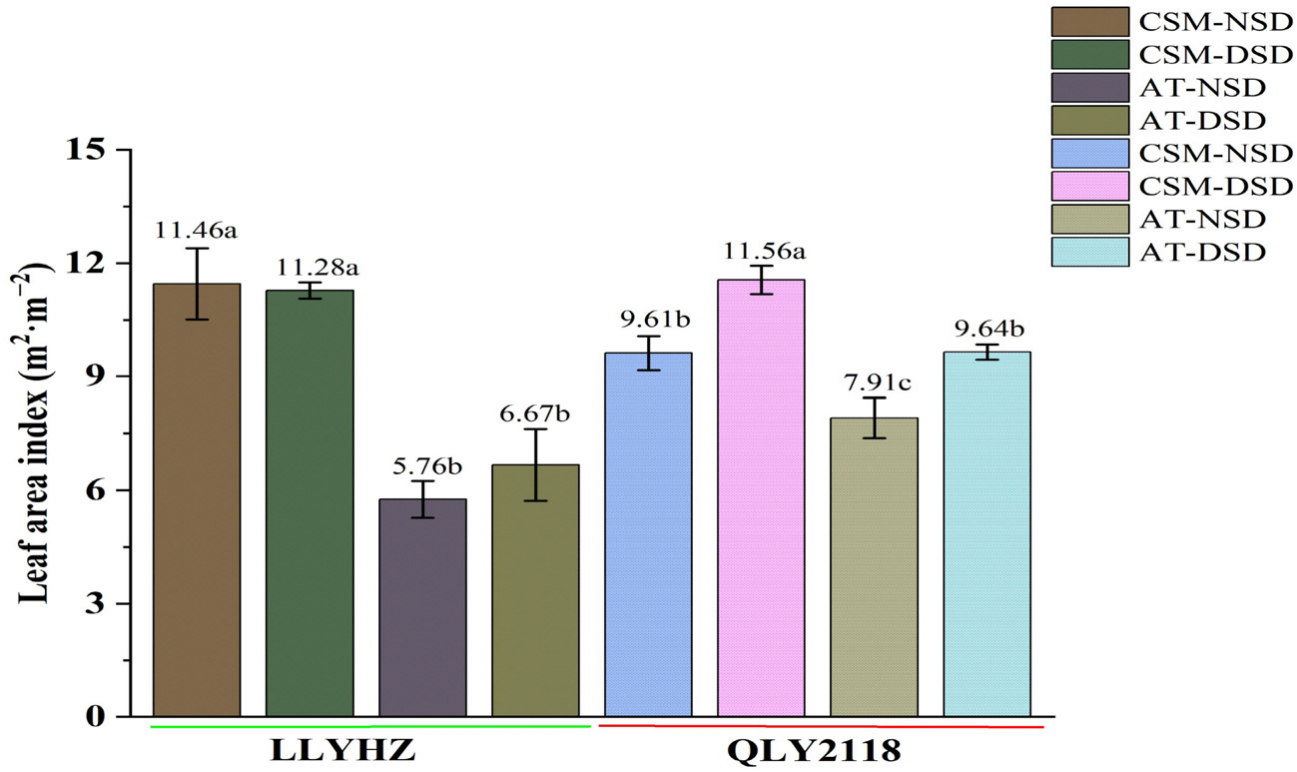
Leaf area index of two rice cultivars in the different treatments at the heading stage. Error bars indicate the SD of three biological replicates. Bars with different lowercase letters are significantly different (LSD test, *p* < 0.05). LLYHZ, Longliangyou Huazhan; QLY2118, Quanliangyou 2118; NSD, normal sowing date; DSD, delayed sowing date; CSM, carpet seedling mechanical transplantation; MT, manual transplanting; SD, standard deviation.

No significant difference in the leaf area index of LYYHZ was observed between the two sowing dates under the carpet seedling mechanical transplanting treatment [NSD: 11.46 m^2^ m^−2^; delayed sowing date (DSD): 11.28 m^2^ m^−2^] or under the manual transplanting treatment (NSD: 5.76 m^2^ m^−2^; DSD: 6.67 m^2^ m^−2^). The leaf area index of QLY2118 under mechanical transplanting with the normal sowing date (9.61 m^2^ m^−2^) was significantly lower than that with the delayed sowing date (11.56 m^2^ m^−2^). Under the manual transplanting treatment, the leaf area index of QLY2118 with the normal sowing date (7.91 m^2^ m^−2^) was significantly lower than that of the delayed sowing date (9.64 m^2^ m^−2^). Further analysis showed that, with the same sowing date, the leaf area indices of YLLHZ and QLY2118 in the carpet seedling mechanical transplanting treatment were significantly higher than those in the manual transplanting treatment (**Figure 2**).

The canopy temperature of plants at the heading stage under the two planting methods was lower than that of the air temperature, but the difference between the canopy temperature and air temperature under the different planting methods was significant. The canopy temperature of plants during the heading stage in the carpet seedling mechanical transplanting treatment was 0.31–0.63°C lower than the air temperature, whereas the canopy temperature during the heading stage in the manual transplanting treatment was 0.18–0.29°C lower than the air temperature (**Table 1**). These results show that the carpet seedling mechanical transplanting method led to a reduced canopy temperature by increasing the leaf area index.

**Table 1:** Leaf area index, average maximum canopy temperature, and temperature difference between treatments at the heading stage of two rice cultivars.

Cultivar	SD	PM	MMTC (°C)	MMAC (°C)	TDV (°C)
LLYHZ	NSD	CSM	32.51 ± 1.97 a	33.14 ± 2.01 a	0.63
	DSD	CSM	29.71 ± 4.05 b	30.19 ± 4.03 b	0.48
	NSD	AT	33.15 ± 2.04 a	33.34 ± 2.02 a	0.19
	DSD	AT	33.41 ± 2.06a	33.59 ± 2.06 a	0.18
QLY2118	NSD	CSM	33.12 ± 2.16 a	33.43 ± 2.15 ab	0.31
	DSD	CSM	30.74 ± 3.74 b	31.53 ± 3.79 b	0.79
	NSD	AT	33.86 ± 2.22 a	34.01 ± 2.20 a	0.15
	DSD	AT	33.37 ± 1.59 a	33.66 ± 1.59 a	0.29

LLYHZ, Longliangyou Huazhan; QLY2118, Quanliangyou 2118; SD, sowing date; PM, planting method; MMTC, mean maximum canopy temperature; MMAC, mean maximum atmospheric temperature; TDV, temperature difference value; NSD, normal sowing date; DSD, delayed sowing date; CSM, carpet seedling mechanical transplantation; AT, manual transplanting; SD, standard deviation. Different lowercase letters within a column indicate a significant difference (LSD test, p < 0.05). Error values are the SD of three biological replicates.

### 3.3 Grain yield and yield components under the different treatments

A significant interaction was observed between sowing date and planting method on grain yield and percentage seed set. Under the carpet seedling mechanical transplanting treatment, no significant difference in effective panicle number, grain number per panicle, percentage seed set, or grain yield was observed among the sowing date treatments, but the 1,000-grain weight under the normal sowing date (LLYHZ, 187.6; QLY2118, 186) was significantly lower than that under the delayed sowing date (LLYHZ, 231.0; QLY2118, 210.0). Under the manual transplanting method, the grain yield of the normal sowing date treatment (LLYHZ, 11.6 t ha^−1^; QLY2118, 10.6 t ha^−1^) was significantly lower than that of the delayed sowing treatment (LLYHZ, 13.1 t ha^−1^; QLY2118, 13.5 t ha^−1^); the grain yields of LLYHZ and QLY2118 decreased by 11.5% and 21.5%, respectively (**Table 2**).

**Table 2:** Yield of two rice cultivars in the different treatments and their yield components.

Cultivar	SD	PM	NEP (no. m^−2^)	GNP	SSR (%)	TSW (g)	Yield (t ha^−1^)
LLYHZ	NSD	CSM	403.6 ± 17.6 a	187.6 ± 21.0 b	85.2 ± 1.2 a	21.8 ± 0.4 a	16.3 ± 1.0 a
	DSD	CSM	378.1 ± 19.2 a	196.7 ± 13.8 ab	84.3 ± 0.4 ab	22.9 ± 0.2 b	16.8 ± 0.7 a
	NSD	AT	295.9 ± 10.1 b	231.0 ± 34.2 a	80.6 ± 2.0 c	21.6 ± 0.1 bc	11.6 ± 0.4 c
	DSD	AT	311.1 ± 15.1 b	236.2 ± 12.1 a	82.3 ± 1.5 bc	21.3 ± 0.2 c	13.1 ± 0.2 b
QLY2118	NSD	CSM	381.1 ± 21.0 a	186.0 ± 11.4 b	86.0 ± 2.2 a	22.5 ± 0.3 a	14.0 ± 0.7 a
	DSD	CSM	405.8 ± 22.7 a	178.4 ± 5.7 b	87.1 ± 4.1 a	23.8 ± 0.3 b	14.0 ± 0.8 a
	NSD	AT	263.2 ± 22.4 b	210.0 ± 8.5 a	78.3 ± 1.6 b	22.7 ± 0.4 b	10.6 ± 0.4 a
	DSD	AT	289.8 ± 17.7 b	226.7 ± 11.0 a	85.6 ± 0.8 a	22.5 ± 0.3 b	13.5 ± 0.8 b

LLYHZ, Longliangyou Huazhan; QLY2118, Quanliangyou 2118; SD, sowing date; PM, planting method; NSD, normal sowing date; DSD, delayed sowing date; CSM, carpet seedling mechanical transplantation; AT, manual transplanting; NEP, number of effective panicles; GNP, grain number per panicle; SSR, percentage seed set; TSW, thousand-seed weight. Different lowercase letters within a column indicate a significant difference (LSD test, p < 0.05). Error values are the SD of three biological replicates.

### 3.4 Effects of high temperature on percentage seed set and grain quality of pot-grown rice plants

**Figure 3:**
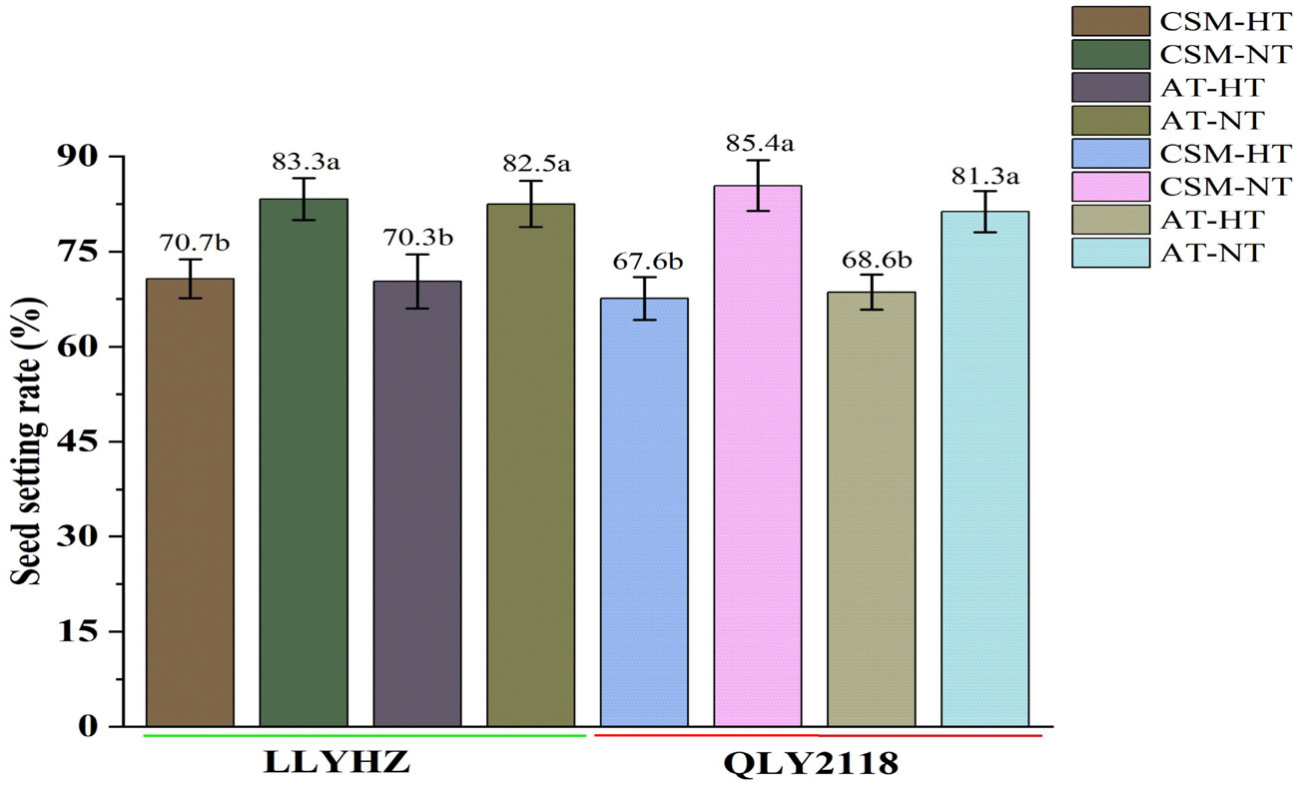
Effect of high temperature on seed set of two rice cultivars under different planting methods. Bars with different lowercase letters indicate a significant difference (LSD test, *p* < 0.05). Error bars indicate the SD of three biological replicates. LLYHZ, Longliangyou Huazhan; QLY2118, Quanliangyou 2118; CSM, carpet seedling mechanical transplantation; AT, manual transplanting; HT, high temperature; NT, normal temperature.

**Figure 4:**
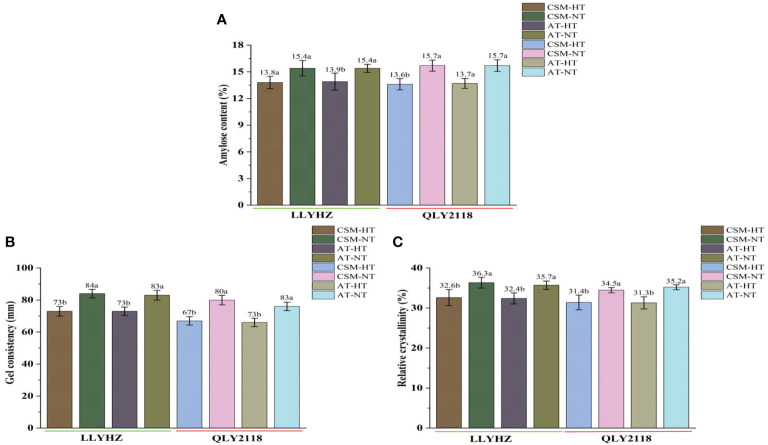
Effect of high temperature on grain quality of two rice cultivars. **(A)** Amylose content of the grains. **(B)** Gel consistency of the grains. **(C)** Relative crystallinity of the grains. Bars with different lowercase letters indicate a significant difference (LSD test, *p* < 0.05). Error bars indicate the SD of three biological replicates. LLYHZ, Longliangyou Huazhan; QLY2118, Quanliangyou 2118; CSM, carpet seedling mechanical transplantation; AT, manual transplanting; HT, high temperature; NT, normal temperature.

High temperature had a significant effect on the percentage seed set under both planting methods (**Figure 3**). Under carpet seedling mechanical transplantation (70.7%) and manual transplanting (70.3%), the percentage seed set of LLYHZ under high-temperature treatment was 15.1% and 14.8% lower than that under normal temperature treatment (83.3% and 82.5%), respectively, and the percentage seed set of QLY2118 under high-temperature treatment (67.6% and 68.6%) was 20.8% and 15.6% lower than that under normal temperature treatment (85.4% and 81.3%), respectively. Under exposure to high-temperature stress, grain quality decreased significantly. High temperature significantly decreased the amylose content, gel consistency, and relative crystallinity of the grain under both transplanting methods (**Figures 4A–C**).

### 3.5 Effects of plant growth regulators on anther dehiscence and percentage seed set

**Figure 5:**
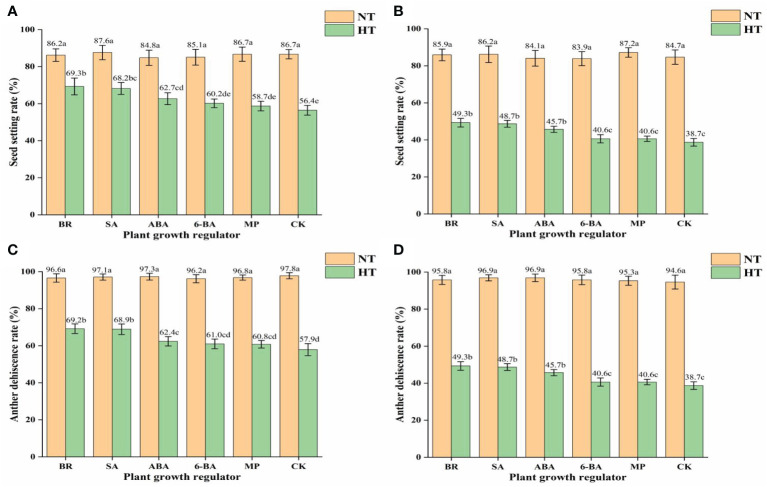
Effects of plant growth regulators on seed set and anther dehiscence of two rice cultivars under different temperature treatments. **(A)** Seed set of Huanghuazhan (HHZ). **(B)** Seed set of Huiliangyou 858 (HLY858). **(C)** Anther dehiscence of HHZ. **(D)** Anther dehiscence of HKY858. Bars with different lowercase letters indicate a significant difference (LSD test, *p* < 0.05). Error bars indicate the SD of three biological replicates. HT, high temperature; NT, normal temperature. BR, brassinolide; SA, salicylic acid; ABA, abscisic acid; 6-BA, benzylaminopurine; MP, potassium dihydrogen phosphate; CK, water.

High-temperature treatment had a significant effect on the percentage seed set (**Figures 5A, B**). The percentage seed set of HHZ sprayed with BR, SA, ABA, 6-BA, MP, and CK was 19.6%, 22.1%, 26.1%, 29.3%, 32.2%, and 53.7%, respectively. The percentage seed set of HLY858 sprayed with BR, SA, ABA, 6-BA, MP, and CK was 42.6%, 43.5%, 45.7%, 51.6%, 53.4%, and 54.3%, respectively. The decrease in seed set under high-temperature treatment of the heat-sensitive cultivar HLY858 was greater than that of the heat-resistant cultivar HHZ. Under high-temperature treatment, compared with the water spray treatment, spray application of growth regulators increased the percentage seed set. Different growth regulators had different effects on alleviating high-temperature stress. The CK, BR, and SA treatments had the strongest effect on increasing the percentage seed set.

High-temperature treatment had a significant effect on anther dehiscence (**Figure 5C, D**). Compared with the normal temperature treatment, the average anther dehiscence percentage of HHZ and HLY858 decreased by 34.5% and 54.2%, respectively, under high-temperature stress. Under the high-temperature treatment, compared with the control, spray application of growth regulators increased the anther dehiscence percentage. The growth regulators differed in their effect on alleviation of high-temperature stress, of which brassinolide and salicylic acid treatment had the greatest effect on increasing the anther dehiscence percentage.

### 3.6 Effects of plant growth regulators on *the* antioxidant enzyme activities of rice spikelets

**Figure 6:**
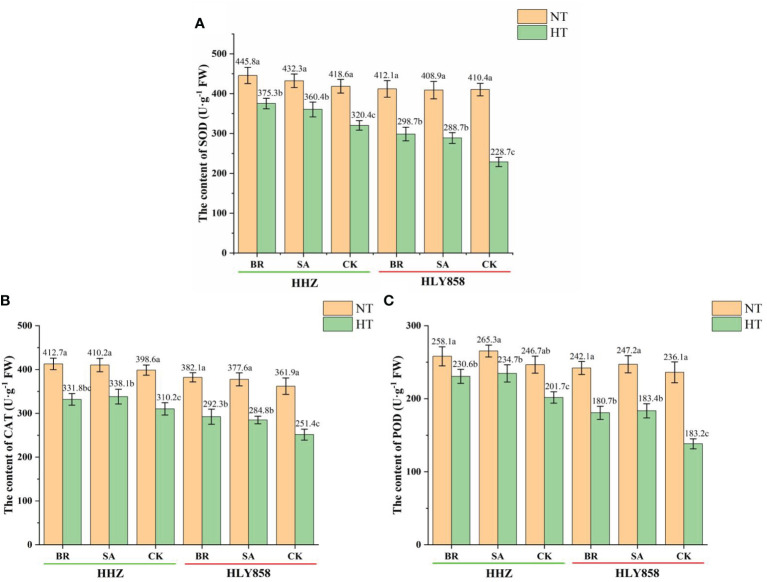
Effects of plant growth regulators on alleviating high temperature stress on antioxidant enzymes in the spikelet of two rice cultivars. **(A)** Superoxide dismutase (SOD); **(B)** catalase (CAT); **(C)** peroxidase (POD). Bars with different lowercase letters indicate a significant difference (LSD test, *p* < 0.05). Error bars indicate the SD of three biological replicates. HHZ, Huanghuazhan; HLY858, Huiliangyou 858; HT, high temperature; NT, normal temperature; BR, brassinolide; SA, salicylic acid; ABA, abscisic acid; 6-BA, 6-benzylaminopurine; MP, potassium dihydrogen phosphate; CK, water.

High-temperature treatment had a significant effect on the antioxidant enzyme activity of rice spikelets (**Figures 6A–C**). Compared with the normal temperature, the SOD activity in spikelets of HHZ sprayed with BR, SA, and CK under high-temperature treatment decreased by 15.8%, 16.6%, and 23.5%, respectively, whereas that of HLY858 under high-temperature treatment decreased by 27.5%, 29.4%, and 44.3%, respectively. Compared with the normal temperature, the POD activity in spikelets of HHZ sprayed with BR, SA, and CK under high-temperature treatment decreased by 10.7%, 11.5%, and 18.2%, respectively, and that of HLY858 under high-temperature treatment decreased by 25.4%, 25.8%, and 41.5%, respectively. Compared with the normal temperature, the CAT activity in spikelets of HHZ sprayed with BR, SA, and CK under high-temperature treatment decreased by 19.6%, 17.5%, and 22.1%, and that of HLY858 under high-temperature treatment decreased by 23.5%, 24.6%, and 30.5%, respectively. The decrease in antioxidant enzyme activities of the heat-sensitive cultivar HLY858 was significantly greater than that of the heat-tolerant cultivar HHZ. Under high-temperature treatment, compared with the water treatment, spray application of growth regulators increased the activity of antioxidant enzymes in the two rice cultivars. The increase in activities of HLY858 was greater than that in HHZ.

## 4 Discussion

### 4.1 Effects of high temperature on *the* yield and quality of rice

The formation of crop yield is the process by which crops use environmental resources to complete their growth cycle to maturity and develop economically valuable products. Temperature is arguably the most easily monitored meteorological indicator among the many environmental factors that consistently affect yield. The components of rice yield include panicle number, grain number, percentage seed set, and 1,000-grain weight. The temperature requirements differ among the developmental stages of rice. Previous studies have shown that high-temperature stress in rice leads to pollen swelling and deformation (Wu et al., 2017) and ‘sticky’ pollen grains that adhere together in the anther, ultimately leading to a reduced frequency of pollen germination on the stigma (Rativa et al., 2020). In addition, high temperature at the heading stage affects the maturation and germination of pollen grains, reduces the percentage fertilization, and increases the number of empty grains, resulting in decreases in seed set and 1,000-grain weight (Satake et al., 1988). With regard to yield components in rice, the optimum temperature in the vegetative growth period is 28.4°C, and during the grain-filling period it is 21.7–26.7°C; the daily average maximum temperature should not exceed 35°C. If the temperature exceeds 27°C during the grain-filling period, it will cause a reduction in the percentage seed set and 1,000-grain weight and will affect the grain yield. This is consistent with the present results, which show that both natural high temperature and simulated high temperature reduced the percentage seed set. High temperature also affects the grain quality of rice. High temperature during the grain-filling stage leads to a reduction in the gel consistency and amylose content of the grain (Lin et al., 2010). Kobata et al. (Kobata et al., 2004) reported that high temperature leads to a lack of starch substrates in the endosperm and an increase in grain chalkiness, and the abnormal loss of endosperm moisture under high temperature also leads to the development of chalkiness (Ishimaru et al., 2009). Consistent with previous studies, the present results show that high temperature significantly reduced the amylose content, gel consistency, and relative crystallinity of rice grains, confirming that high temperature reduces the grain quality and yield of rice.

### 4.2 Reasonable sowing date to alleviate *high-temperature* damage *to* rice

One means of avoiding the risk of high-temperature stress is to adjust the sowing date to stagger the crucial stages of rice growth and the timing of high temperature to alleviate the effects of high temperature on rice. Selection of an appropriate sowing date is conducive to efficient development of the production potential in rice. In the present experiment, rice grown under a normal sowing date was more likely to suffer from high-temperature stress and significant decreases in the percentage seed set and grain yield than rice grown under a delayed sowing date. A delayed sowing date effectively avoided the effects of high temperature in the heading and flowering stages, which was beneficial to the growth of the plant. However, the delay in sowing date shortens the vegetative growth period, and the population growth is reduced, which negatively affects the yield components and results in lower yield (Patel et al., 2019). An excessively early or overly late sowing date is not conducive to the coordinated development of rice plants (Kumar et al., 2012). In addition, the sowing date has a strong impact on grain quality. High temperature at the heading stage increases the amylose and protein contents and decreases the gel consistency of the grain (Krishnan et al., 2011). However, the decrease in amylose content under high temperature may reflect genotypic differences among cultivars (Sreenivasulu et al., 2015). The sowing date can alter the severity of damage of high temperature on rice, change the temperature and light resources during the grain-filling period, and indirectly or directly affect grain quality and yield. An early sowing date and short growth period are not conducive to most effectively utilizing the local temperature and light environment. Late-maturing cultivars with a relatively long growth period can be chosen to exploit local temperature and light resources. Late sowing of cultivars with a long growth period will affect the reliability of full heading and late grain filling, and the cultivar’s yield potential will not be realized. Therefore, the selection of a cultivar with a slightly shorter growth period, rapid tillering, and favorable traits for early maturation will help to ensure grain filling and maturity and that a high yield is attained.

### 4.3 Alleviating heat injury *in* rice by different planting methods

Given differences in the utilization efficiency of resources, such as temperature and light, different planting methods will inevitably have a certain impact on the growth of rice. Differences were observed in the adaptability of rice to high temperatures in summer under different planting methods. For single-season indica rice cultivars with an initial sowing period of more than 94 d in the Jianghuai area of Anhui Province, the mechanical transplanting of carpet seedlings (sown on 16 May) effectively avoided high-temperature stress at the heading and flowering stages. In addition, carpet seedling mechanical transplantion can alleviate the damage from high temperature to a certain extent by increasing population growth and reducing the canopy temperature. The yield of rice under different planting methods was previously reported to be significantly positively correlated with the accumulation of total dry matter (San-Oh et al., 2008). The material output rate and conversion rate of rice leaves and stems are highest in hand-transplanted rice and lowest in direct-seeded rice.

The damage from high temperature differs among rice seedlings grown under different planting methods, which affects the entire development process. The duration of the seedling stage is used to avoid the risk of high-temperature exposure at a later developmental stage. In addition, the yield under different planting methods also differs. Thus, choosing the most appropriate planting method is crucial to maximize the potential benefits.

### 4.4 Chemical regulation alleviates high-temperature damage to rice

The application of exogenous plant growth regulators can reduce damage from high temperature. Mixed application of plant growth regulators under high temperature can increase the photosynthetic capacity of rice leaves and improve spikelet fertility and grain filling (Fahad et al., 2016b). Spray application of exogenous ABA may regulate stomatal closure in the leaves, induce the expression of responsive genes and the synthesis of endogenous ABA and heat shock proteins, and improve the high-temperature tolerance of plants (Rezaul et al., 2019). Spray application of brassinolide in the middle and late stages of pollen mother cell meiosis increases SOD activity and the contents of proline, soluble sugar, and ABA in florets at the heading stage, improves the high-temperature tolerance of plants at the heading stage, and reduces the decrease in percentage seed set under high-temperature stress at the heading stage (Chen et al., 2021; Raghunath et al., 2021). In addition, spray application of SA (Yang et al., 2022), auxin (Sharma et al., 2018), MP (Yang et al., 2019), and other substances can alleviate high-temperature stress to a certain extent. In the present study, spray application of five plant growth regulators effectively reduced damage from high temperatures on rice. The main responses were to improve SOD, POD, and CAT activities, percentage anther dehiscence, and percentage seed set. Among the growth regulators applied in this study, brassinolide and SA had the strongest beneficial effects.

### 4.5 Simplified green cultivation system for single-season indica rice adapted to high-temperature stress in the middle and lower reaches of the Yangtze River

Based on physiological and agronomic research on high-temperature resistance, it is very important to optimize the existing cultivation techniques and regulate the growth and development process of rice. These are useful in making rice plants adapt to the high-temperature stress caused by global warming. This study integrated high-temperature damage and control technology models for indica rice in the middle and lower reaches of the Yangtze River. The conclusions are as follows: (1) select the varieties with high quality, high yield, and high-temperature resistance; (2) determine a reasonable sowing date to avoid high temperature during the heading and flowering stages of rice; (3) mechanize rice transplanting to establish a healthy community structure and improve rice resistance to high temperature; and (4) compensate with cultivation disaster reduction measures (i.e., application of plant growth regulators).

## 5 Conclusion

The present results show that carpet seedling mechanical transplantation and a delayed sowing date in combination effectively avoided high-temperature stress at the heading and flowering stages of rice. The carpet seedling transplanting method promoted population growth, thereby reducing the canopy temperature and alleviating the risk of high-temperature stress. However, high temperatures led to a decrease in the percentage seed set and reduced grain quality under both mechanical transplanting and manual transplanting. The plant growth regulators brassinolide and SA alleviated injury from high-temperature stress at the heading and flowering stages by increasing the activity of antioxidant enzymes in the panicles, thereby reducing the damage caused by accumulation of reactive oxygen species, enhancing the high-temperature tolerance of plants, and increasing the percentage of anther dehiscence and the percentage seed set.

## Data availability statement

The original contributions presented in the study are included in the article/**Supplementary Material**. Further inquiries can be directed to the corresponding author.

## Author contributions

MZ, ZL, and YZ: Conceptualization. MZ, ZL, KF, BT, QL, and YZ: investigation; MZ, YJ, YX, JL, DT, and WW: formal analysis. MZ, ZL, and YZ: writing. All authors have read and agreed to the published version of the manuscript.

## Glossary

**Table:**

LLYHZ	Longliangyou Huazhan
QLY2118	Quanliangyou 2118
HHZ	Huanghuazhan
HLY858	Huiliangyou 858
AT	Artificial transplanting
CSM	Carpet seedlings machine
SD	Sowing date
PP	Planting pattern
DSD	Delaying sowing date
NSD	Normal sowing date
TEP	The effective panicles
GNP	Grain number per panicle
SSR	Seed setting rate
TSW	Thousand seed weight
SOD	Superoxide dismutase
POD	Peroxidase
CAT	Catalase
BR	Brassinolide
SA	Salicylic acid
ABA	Abscisic acid
6-BA	6-benzylaminopurine
MP	Monopotassium phosphate
MMTC	Mean maximum canopy temperature
MMAC	Mean maximum atmospheric temperature
TDV	Temperature difference value
