# Supplementary material for: Strategies for indica rice adapted to high-temperature stress in the middle and lower reaches of the Yangtze River
Source: Front Plant Sci. 2023 Jan 6;13:1081807. doi: 10.3389/fpls.2022.1081807 (PMC9852850; doi:10.3389/fpls.2022.1081807)
Supplement: Supplementary file 1 [file Table_1.doc]

**Table S1 Days of high temperature at heading and flowering stage under different treatments**

| Variety | SD | PP | HTD | Daily maximum temperature (℃) | | | | | | | | | | | | | |
| --- | --- | --- | --- | --- | --- | --- | --- | --- | --- | --- | --- | --- | --- | --- | --- | --- | --- |
|  |  |  |  | 7-DBH | 6-DBH | 5-DBH | 4-DBH | 3-DBH | 2-DBH | 1-DBH | 1-HD | 2-HD | 3-HD | 4-HD | 5-HD | 6-HD | 7-HD |
| LLYHZ | NSD | CSM | 1 | 35.2 | 33.2 | 32.6 | 34.0 | 34.2 | 32.5 | 34.4 | 33.5 | 34.0 | 34.9 | 34.6 | 27.3 | 31.4 | 32.2 |
|  | DSD | CSM | 0 | 34.4 | 33.5 | 34.0 | 34.9 | 34.6 | 27.3 | 31.4 | 32.2 | 30.3 | 25.0 | 23.1 | 24.7 | 28.8 | 28.5 |
|  | NSD | AT | 3 | 29.0 | 29.6 | 32.8 | 35.4 | 33.9 | 33.7 | 36.2 | 35.2 | 33.2 | 32.6 | 34.0 | 34.2 | 32.5 | 34.4 |
|  | DSD | AT | 2 | 33.7 | 36.2 | 35.2 | 33.2 | 32.6 | 34.0 | 34.2 | 32.5 | 34.4 | 33.5 | 34.0 | 34.9 | 34.6 | 27.3 |
| QLY2118 | NSD | CSM | 2 | 36.2 | 35.2 | 33.2 | 32.6 | 34.0 | 34.2 | 32.5 | 34.4 | 33.5 | 34.0 | 34.9 | 34.6 | 27.3 | 31.4 |
|  | DSD | CSM | 0 | 34.0 | 34.2 | 32.5 | 34.4 | 33.5 | 34.0 | 34.9 | 34.6 | 27.3 | 31.4 | 32.2 | 30.3 | 25.0 | 23.1 |
|  | NSD | AT | 8 | 34.0 | 35.8 | 35.3 | 35.2 | 35.0 | 35.1 | 29.0 | 29.6 | 32.8 | 35.4 | 33.9 | 33.7 | 36.2 | 35.2 |
|  | DSD | AT | 3 | 29.6 | 32.8 | 35.4 | 33.9 | 33.7 | 36.2 | 35.2 | 33.2 | 32.6 | 34.0 | 34.2 | 32.5 | 34.4 | 33.5 |

Note: SD: Sowing date. PP: Planting pattern. HTD: The number of days when the temperature at heading and flowering was higher than 35.0℃. DBH: Days before heading. HD：Days of heading. NSD: Normal sowing date. DSD：Delaying sowing date. CSM: Carpet seedlings machine. AT: Artificial transplanting.
